# Special Issue “Cancer Biomarker Research and Personalized Medicine 2.0”

**DOI:** 10.3390/jpm14060549

**Published:** 2024-05-21

**Authors:** James Meehan, Mark Gray

**Affiliations:** The Royal (Dick) School of Veterinary Studies and Roslin Institute, University of Edinburgh, Easter Bush, Roslin, Midlothian, Edinburgh EH25 9RG, UK; mark.gray@ed.ac.uk

In 2022, there was an estimated incidence of 20 million cancer cases and 9.7 million deaths from cancer worldwide. By the year 2050, the rates of cancer incidence and death are projected to increase to 35.3 and 18.5 million, respectively [1]. It is thought that cancer mortality rates will surpass cardiovascular disease mortality rates in the near future [2]. While improvements have been made in the diagnosis and treatment of various cancer types, it is evident from these statistics that further breakthrough advancements are necessary to decrease the significant social and economic impact this disease has on the population.

Personalized medicine is the use of tailored treatments for individual patients, and represents a departure from conventional, generalized medical approaches. While personalized medicine has applications across a range of medical disciplines, its use in the field of oncology perhaps holds the most promise. Cancer, being a heterogeneous disease, exhibits significant variability in genetic mutations, molecular characteristics and tumour microenvironments between patients diagnosed with the same type of cancer. This heterogeneity is a crucial characteristic of cancer, greatly impacting prognosis and treatment efficiency, and is perhaps one of the greatest challenges confronting scientists and clinicians today [3].

Biomarkers are biological markers through which a particular physiological/pathological process or disease can be identified. Cancer biomarkers encompass a wide array of tissue and liquid-based biochemical substances, including DNA, RNA, intracellular/secreted proteins, extracellular vesicles and circulating tumour cells. Over recent decades, there has been a significant push towards achieving personalized cancer treatment through the discovery and use of cancer-specific biomarkers [4].

This Special Issue entitled ‘Cancer Biomarker Research and Personalized Medicine 2.0’ contains a collection of research articles focusing on biomarkers in a variety of cancer types. In this Editorial, we give a brief summary of the key findings from each article, while also drawing attention to the wider implications of this research for advancing the application of personalized medicine for cancer patients.

Breast cancer is the most commonly diagnosed female neoplasm in the world [5]. Conventional histological classification systems are widely used to provide a diagnosis and select the most appropriate treatments for these patients [6]. The use of targeted therapies, for example, is based on the presence of cellular receptors such as oestrogen receptor and human epidermal growth factor receptor 2 [7]. More recently, the integration of molecular markers into breast cancer classification systems has allowed for further treatment selection guidance and the identification of novel therapeutic targets. In this Special Issue, Erdogdu et al. [8] investigated the relationship between menopausal status and the histological, molecular and somatic mutation profiles of breast cancer patients. Using next-generation sequencing technology, this interesting paper demonstrated that 94% of both premenopausal and postmenopausal breast cancer patients harboured somatic mutations in established cancer susceptibility genes including *TP53*, *PIK3CA*, *BRCA2*, *NF1*, *PTEN*, *ATR*, *CHEK2*, *BLM*, *BRAC1*, *PMS2* and *ATM*. The authors suggested that these results contribute to the understanding of the pathogenesis of breast cancer in relation to menopausal status. Furthermore, due to the high prevalence of genetic mutations, the authors also propose that genetic testing could be used to improve treatment selection for premenopausal and postmenopausal patients.

Another common neoplasia is colorectal cancer. This cancer type is the third most common cancer and the second leading cause of cancer-related deaths in the world [9]. Early diagnosis with timely and appropriate treatment selection could improve survival rates. One way in which this could be achieved is through the use of prediction models to identify symptomatic patients with higher colorectal cancer risk for whom referral is most appropriate [10]. These types of models could assist clinical care decision-making, including risk-tailored cancer screening, testing and treatment. In this Special Issue, Xu et al. [11] developed and validated prediction models incorporating demographics, clinical features and a weighted genetic risk score to predict colorectal cancer risk in symptomatic patients. Their findings suggested that the integration of genetic factors into prediction models improved their performance. The authors proposed that this could help to identify symptomatic patient cohorts with higher colorectal cancer risk due to genetic susceptibility. The authors are now pursuing external model validation and investigation of its potential clinical impact.

Staying on the topic of colorectal cancer, Alsalman et al. [12] investigated whether complete blood count parameters were associated with prognosis. Using clinicopathologic features including tumour budding, disease stage and tumour anatomical location, complete blood count parameters were compared to disease-free survival. The authors demonstrated that regardless of tumour anatomical location, higher mean platelet volumes and lower eosinophil numbers were present in early-stage disease and could serve as potential prognostic biomarkers. Additionally, lower levels of mean platelet volume, mean corpuscular haemoglobin concentration and haemoglobin, and high levels of eosinophils and red cell distribution width were associated with shorter disease-free survival in left-sided colorectal cancer patients. In contrast, higher platelet levels were associated with worse disease-free survival in the right-sided colorectal cancer patients. The authors proposed that some complete blood count parameters could be useful for predicting disease-free survival in pre-treatment patients, with the advantage that this test could be widely utilised in daily clinical practice as it is routinely available, simple and inexpensive. They suggested that their results should be validated in larger cohorts with longer follow-up times to include overall survival.

Investigating a rarer cancer type, Modica et al. [13] performed research into medullary thyroid cancer biomarkers. Originating from parafollicular cells of the thyroid gland, this neoplasm is a rare neuroendocrine neoplasm accounting for <5% of thyroid cancers [14]. Due to disease rarity and often indolent clinical course, there is a need for reliable diagnostic biomarkers. Calcitonin is a clinically used biomarker for this disease; however, limitations associated with it include its rapid degradation, inter-assay variation and increased levels due to other diseases [15,16]. This study investigated the potential role of neutrophil-to-lymphocyte ratio (NLR), platelet-to-lymphocyte ratio (PLR) and systemic immune–inflammation index (SII) as medullary thyroid cancer biomarkers. Using clinical and histology data, calcitonin, NLR, PLR and SII levels were analysed before and after thyroidectomy. Although statistically significant differences in NLR, SII and calcitonin levels were observed following thyroidectomy, there was no association between their values, prognosis or tumour characteristics. However, the authors proposed that elevated pre-operative NLR and SII might indicate the presence of disease-associated inflammation, which could be related to tumour growth. The authors suggested that further studies are needed to define their roles as prognostic or diagnostic medullary thyroid cancer biomarkers.

Extracellular vesicles (EVs) are released into the systemic circulation and can be isolated from routine blood and urine samples. EVs are thought to have a crucial role in cancer development, and are believed to have great potential as liquid-based biomarkers for cancer diagnosis and management [17]. However, despite this interest in their use for personalized medicine, no EV biomarker has yet entered clinical practice. Here, Di Santo et al. [18] developed a novel approach for the characterisation of EVs. In this pilot study, the authors investigated the capability of Fourier Transform Infrared (FTIR) spectroscopy to characterise and differentiate EVs acquired from patients diagnosed with hepatocellular carcinoma of non-viral origin and EVs from control patients. Based on their mid-IR spectral response, differences in the carbohydrate and nucleic acid band, the protein amide I and II bands, and the lipid CH stretching band were identified. Analysis suggested that these spectral biomarkers can outperform two widely used hepatocellular carcinoma biomarkers (alpha-fetoprotein and protein induced by the absence of vitamin K or antagonist-II [PIVKA-II]). This exciting study provided proof-of-concept that label-free EV molecular profiling, using FTIR spectroscopy, is a possible method for cancer diagnosis.

The five papers contained within this Special Issue give an insight into the wide range of biomarker research that is being performed within the scientific community. With exciting research areas such as these, we believe that in the years to come, novel biomarkers will become more commonly used in clinical practice, helping to achieve a more personalized approach to cancer patient care.
